# Echocardiographic parameters of systemic right ventricular size and function compared to cardiac MRI

**DOI:** 10.1186/1532-429X-16-S1-P130

**Published:** 2014-01-16

**Authors:** Emily Ruckdeschel, Jeremy Nicolarsen, Elizabeth Yeung, Amber Khanna, Joseph Kay

**Affiliations:** 1 Pediatric Cardiology, Childrens Hospital Colorado, Aurora, Colorado, USA; 2 Cardiology, University of Colorado, Aurora, Colorado, USA

## Background

Patients with dextro-Transposition of the Great Arteries (d-TGA) who have undergone atrial switch palliation and patients with congenitally corrected Transposition of the Great Arteries (ccTGA) have systemic right ventricles which are prone to long term dysfunction and failure. Assessment of right ventricular function by echocardiography has been challenging because of anatomical location and shape. Quantitative echocardiographic measurements have been validated in sub-pulmonary right ventricles, but are understudied in systemic right ventricles. Cardiac MRI (cMRI) is the current gold standard in RV assessment but is time consuming and expensive.

## Methods

27 patients with either d-TGA (n = 15) or ccTGA (n = 12) who had both a cMRI and echocardiogram within 6 months of each other were evaluated. Echocardiographic parameters of RV size including measurements at the base, mid cavity, longitudinal axis, and end diastolic area were compared to volume measurements made by cMRI. Echocardiographic parameters of function including myocardial performance index (MPI), dP/dt and fractional area change (FAC) were compared to cMRI measurements of ejection fraction. Statistical analysis was done using linear regression analysis comparing echocardiographic parameters to cMRI results in all patients in the study and in the subgroups of d-TGA and ccTGA.

## Results

Echocardiographic measurements of RV size in the longitudinal axis, base, and end diastolic area were predictive of RV end-diastolic volume (RVEDV) as measured by cMRI (r2 = .55 p =.0001, r2 = .3 p =.0044, r2 = .45 p =.003 respectively) in the combined group of d-TGA and ccTGA. MPI, FAC and dP/dt were not predictive of RV ejection fraction (RVEF) by cMRI in the combined group of d-TGA and l-TGA. dP/dt was predictive of RVEF in patients with ccTGA (r2 = .47, p = .0203).

## Conclusions

Echocardiographic measurements of RV size including longitudinal and base measurements, and end diastolic area were predictive of RVEDV as measured by cMRI in patients with systemic right ventricles. dP/dt was predictive of RVEF determined by cMRI in patients with ccTGA.

## Funding

None.

**Figure 1:**
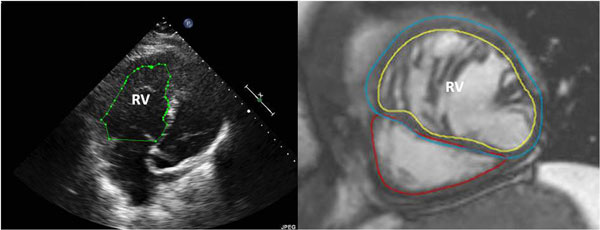
**Measurement of right ventricular (RV) end diastolic area by echocardiogram (panel 1) and measurement of RV end diastolic volume by cardiac MRI (panel 2)**.

